# Concurrent Diagnoses of Cutaneous Sarcoidosis and Recurrent Metastatic Breast Cancer: More than a Coincidental Occurrence?

**DOI:** 10.1155/2018/2812439

**Published:** 2018-09-05

**Authors:** Jacqueline Deen, Nick Mellick, Laura Wheller

**Affiliations:** ^1^Department of Dermatology, Sunshine Coast University Hospital, 6 Doherty Street, Birtinya, Queensland 4575, Australia; ^2^Department of Pathology, Medlab Pathology, 280 Newmarket Road, Wilston, Queenslan 4051, Australia; ^3^Department of Dermatology, Mater Misercordiae Hospital, Raymond Terrace, South Brisbane, Queensland 4101, Australia

## Abstract

Sarcoidosis is a rare, chronic, multisystem disease of unknown aetiology, characterised by non-caseating epithelioid cell granulomas. Its association with internal malignancy, in particular haematological cancers has been strongly documented in the literature, while its link with solid organ malignancies is less extensively reported. We present an atypical case of cutaneous sarcoidosis occurring in association with breast cancer recurrence in a 49-year-old female. Physician recognition of this link between sarcoidosis and internal malignancy is vital because many cases of sarcoidosis in association with neoplasia present initially, or even exclusively, with cutaneous sarcoidal lesions that may precede the development of cancer by several years, or as in our case, present as a cutaneous marker of concomitant underlying malignancy. Our case highlights the importance of age-appropriate cancer screening in additional to a routine work-up for systemic sarcoidosis in a patient with cutaneous sarcoidosis.

## 1. Introduction

Sarcoidosis is a chronic, idiopathic, multisystem disease, characterised by non-caseating epithelioid cell granulomas. The lungs are involved in more than 90% of cases, but the lymphatic system, eyes, and skin may also be affected. Less common but usually more severe forms can involve the liver, spleen, central nervous system, heart, upper respiratory tract, and bones. Its pathogenesis appears to correspond to an aberrant immune response in a susceptible host. Sarcoidosis typically affects young adults, with a slight female general predominance [1, 2].

Various diseases have been associated with sarcoidosis, including autoimmune disorders such as rheumatoid arthritis, psoriasis, vasculitis, thyroid disease, systemic sclerosis, and Sjogren syndrome. Haematological malignancies, in particular lymphoproliferative disorders such as Hodgkin lymphoma, are most strongly associated with sarcoidosis compared to solid organ malignancies [2, 3]. We present a rare case of cutaneous sarcoidosis occurring in association with breast carcinoma.

## 2. Case Report

A 49-year-old female presented with a 2-month history of asymptomatic lesions on the left knee found incidentally on routine full skin examination. The patient was otherwise well, with no pulmonary or systemic symptoms.

She had a past history of breast cancer diagnosed 4 years ago, managed by lumpectomy and adjuvant chemoradiotherapy achieving remission. The patient had regular cancer surveillance and was currently on adjuvant tamoxifen, with a planned duration of 10 years. Her other notable medical history included lifelong asthma, gastrooesophageal reflux disease, depression, subacute thyroiditis and previous shoulder, and knee arthroscopies. Her regular medications included tamoxifen, pantoprazole, venlafaxine, budesonide/formoterol, and terbutaline. She was a lifetime non-smoker and rarely consumed alcohol. The patient had no family history of autoimmune conditions.

**Figure 1:**
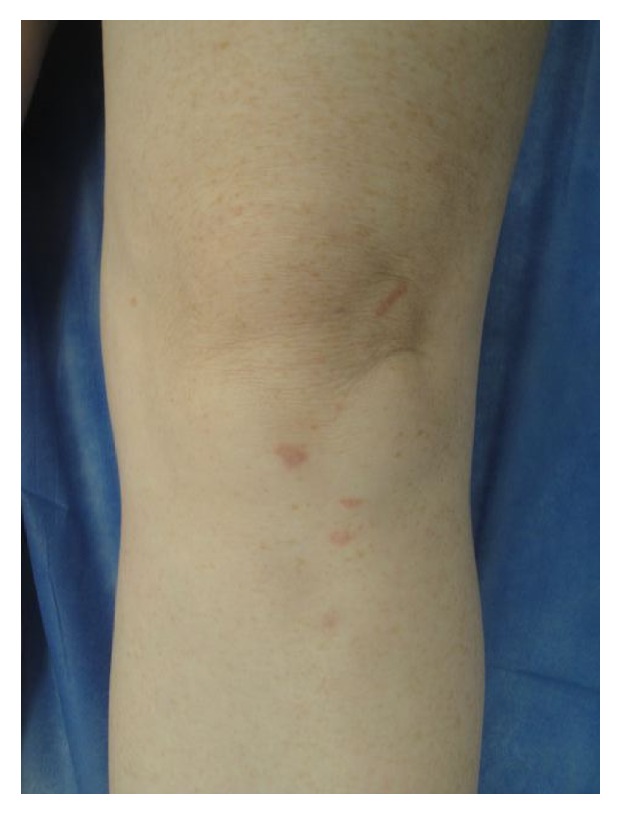
Clinical photograph showing numerous erythematous-to-brown papules on the anterior left knee.

Examination revealed numerous erythematous-to-brown, non-tender papules occurring on the anterior left knee (Figure 1). On the right foot, at the site of a scar from prior cryotherapy for plantar warts, the patient had a similar area of firm indurated erythematous-to-brown change. Dermoscopy of both sites showed orange and yellow translucent globules (“apple-jelly” sign). There were no skin lesions detected on full skin examination suspicious for malignancy. There was no lymphadenopathy and systemic examination was otherwise unremarkable.

**Figure 2:**
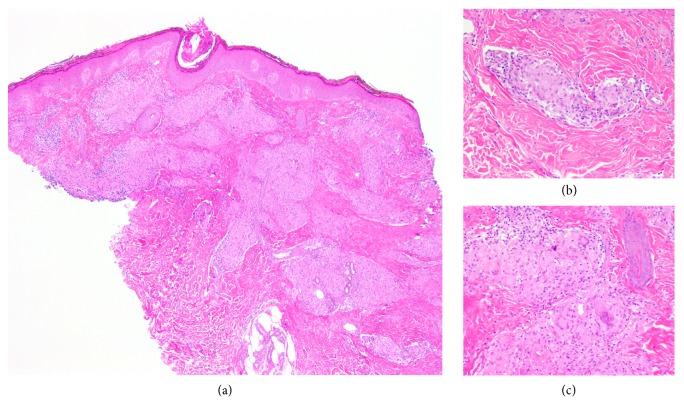
(a, b and c) Histology of left knee lesion showing a sarcoidal-type granulomatous reaction. (a) Much of the reticular dermis is occupied by a granulomatous infiltrate (hematoxylin and eosin staining, original magnification x40). (b and c) Individual granulomas are sarcoidal in type, i.e., non-necrotising with a minimal associated lymphocytic infiltrate (so-called “naked” granulomas) (hematoxylin and eosin staining, original magnification x200).

Skin biopsy showed multiple, variably sized naked sarcoidosis type granulomas scattered throughout the dermis (Figure 2). Chest radiograph showed bilateral hilar lymphadenopathy and serum angiotensin-converting enzyme was elevated at 107 U/L. Other laboratory tests were within normal limits (full blood count, liver and renal function tests, and calcium and inflammatory markers). Further investigations excluded systemic sarcoidosis (cardiac MRI and CT-PET scan). The CT PET ordered during systemic work-up, however, showed a solitary lesion in the T10 vertebra and subsequent biopsy proved recurrent metastatic breast cancer.

The patient's management was then deferred to a medical oncologist for ongoing care of her metastatic breast cancer. She received stereotactic radiation to her spinal lesion and was commenced on a special access program with ribociclib. Following breast cancer treatment, cutaneous sarcoidal lesions completely resolved.

## 3. Discussion

Cutaneous involvement presents in 25% of patients with systemic sarcoidosis and may be the only manifestation [4]. Dermatologists are frequently the first clinicians to identify sarcoidosis as specific skin lesions are often the presenting sign and skin biopsy enables early diagnosis. Skin lesions are extremely variable and may be specific or nonspecific. Specific lesions are those that histologically display noncaseating granulomas, which manifest clinically as maculopapules, plaques, lupus pernio, scar-sarcoidosis, and subcutaneous sarcoidosis. Nonspecific lesions lack histological evidence of sarcoid granulomas and the most significant lesions are erythema nodosum. In isolated cutaneous disease, further evaluation is essential as transformation into systemic sarcoidosis occurs in approximately one-third of patients within three years [1, 2].

Various diseases have been associated with sarcoidosis. Previously, an association between sarcoidosis and malignancy has been described, although no clear relationship has been identified. In most cases, sarcoidosis was diagnosed before the detection of an associated neoplasm. Haematological malignancies remain most strongly associated compared to solid tumours [1–3]. Brincker and Wilbek in 1974 were first to describe this association, reporting that, in patients with sarcoidosis, lymphoma occurred 11 times more frequently and lung cancer occurred three times more frequently compared with the general population [5].

Previous literature cases of sarcoidosis occurring with breast cancer are summarised in Table 1. The average patient age was 53 years, with 98.3% being female. In 30 (48.4%) patients the identification of sarcoidosis preceded the diagnosis of breast cancer; in 18 (29.0%) patients breast cancer diagnosis preceded sarcoidosis; and in 14 (22.6%) patients both diseases occurred concomitantly. The average time interval between the diagnosis of sarcoidosis and breast cancer was 8.3 years (range 1-34 years). When breast cancer predated sarcoidosis, the average interval was 4.1 years (range 0.6-12 years). In our case, the patient age at diagnosis was 49 years, which is similar to what was described in the literature.

Our case is unique in that the cutaneous sarcoidosis most likely occurred around the same time the patient's breast cancer recurrence was diagnosed and investigation for systemic sarcoidosis revealed her metastatic disease. This may be an incidental finding or indicate that dysregulation of the immune system mediated by either the breast cancer or sarcoidosis lead to the granulomatous inflammation of sarcoidosis or neoplasm, respectively [3, 4, 34]. In addition, there was complete resolution of the cutaneous sarcoidal lesions following treatment of the patient's metastatic breast cancer, strengthening the correlation between both entities.

Recognition by physicians of this link between sarcoidosis and internal malignancy is vital because many cases of sarcoidosis in association with neoplasia present initially, or even exclusively, with cutaneous sarcoidal lesions that may precede the development of cancer by several years or as in our case, present as a cutaneous marker of concomitant underlying malignancy. Thus, in addition to routine screening for systemic sarcoidosis, patients diagnosed with cutaneous sarcoidosis should be closely followed up, particularly including age-appropriate cancer screening to exclude the development of associated malignancy [1].

## Figures and Tables

**Table 1:** Previous literature cases of breast cancer and sarcoidosis.

Reference	Sex	Patient age (years)	Interval b/t diseases (years)	Sarcoidosis onset*∗*	Tumour type
Prior et al. (1952) [6]	F	59	5	P	Breast adenocarcinoma

Brincker et al. (1974) [5]	F	NS	NS	P	NS
	F			P	
	F			P	
	M			P	

Suen JS et al (1990) [7]	F	50	0.7	A	Breast cancer (stage II)

Shah AK et al (1990) [8]	F	36	3	P	Invasive ductal carcinoma

Von Knorring et al. (1976) [9]	F	74	5	P	Non-metastasizing breast carcinoma

Whittington R et al. (1986) [10]	F	52	0.6	A	Infiltrating ductal carcinoma
	F	42	5	P	Metastatic breast cancer

Reich J et al. (1995) [11]	F	47	10	A	Intraductal breast carcinoma
	F	55	9	P	Infiltrating ductal breast carcinoma

Brechtek B et al. (1996) [4]	F	58	1	P	NS

Seersholm N et al. (1997) [12]	NS	NS	NS	P	NS

Romer FK et al. (1998) [13]	F	Between 19-78 years	NS	P	NS
	F			P	
	F			P	
	F			P	
	F			P	
	F			P	

Askling J et al. (1999) [14]	NS	NS	NS	P	NS
	NS			P	NS

Lower EE et al. (2001) [15]	F	25	5	P	Invasive ductal carcinoma
	F	57	5	P	Infiltrating ductal carcinoma
	F	58	8	P	Invasive ductal carcinoma
	F	40	2	A	Invasive ductal carcinoma
	F	49	1	A	Invasive ductal carcinoma
	F	38	2	A	Invasive ductal carcinoma
	F	36	1	A	Invasive ductal carcinoma
	F	57	8	A	Invasive ductal carcinoma
	F	55	0	C	Invasive ductal carcinoma
	F	43	0	C	Intraductal carcinoma

Garcia et al. (2003) [16]	F	44	3	P	Invasive lobular breast carcinoma with ductal and mucinous features

Chen W et al. (2004) [17]	F	NS	NS	P	NS
	F	NS	0	C	NS

Van der Hoeven JJ et al. (2004) [18]	F	NS	0	C	Ductal carcinoma of breast

Gusakova I et al. (2007) [19]	F	69	6	A	Infiltrating ductal carcinoma of breast
	F	60	4	A	Infiltrating ductal carcinoma of breast

Tolaney SM et al. (2007) [20]	F	47	0	C	Invasive lobular carcinoma of breast
	F	51	2	A	Invasive ductal carcinoma
	F	31	0	C	Invasive ductal carcinoma

Ataergin S et al. (2009) [21]	F	75	12	A	Breast cancer (T3N1M0)

Viswanath L et al. (2009) [22]	F	50	2	A	Infiltrating ductal carcinoma breast

Ito T et al. (2010) [23]	F	90	6	A	Metastatic breast cancer
	F	52	4	A	Invasive ductal carcinoma breast

Alexandrescu DT et al. (2011) [1]	F	72	8	P	NS
	F	46	4	P	NS
	F	46	5	P	Infiltrating ductal carcinoma of breast

Bush E et al. (2011) [24]	F	42	0	C	Infiltrating ductal carcinoma of breast

Nishioka M et al (2012) [25]	F	79	0	C	Recurrent breast cancer (local)

DeFilippis EM et al (2013) [26]	F	63	0	C	Stage 1 breast cancer

Akhtari et al. (2014) [27]	F	47	0	C	Ductal invasive carcinoma

Kim et al. (2014) [28]	F	44	2	A	Ductal invasive carcinoma

Zivin et al. (2014) [29]	F	32	0	C	Ductal invasive carcinoma

Altinkaya et al. (2015) [30]	F	70	0	C	Ductal invasive carcinoma

Conte et al. (2015) [31]	F	50	0	C	Ductal invasive carcinoma

El Hammoumi (2015) [32]	F	51	3	A	Lobular carcinoma breast

Chen J et al. (2015) [33]	F	62	7	A	Infiltrating ductal carcinoma
	F	54	0	C	Infiltrating ductal carcinoma
	F	50	24	P	Infiltrating ductal carcinoma
	F	63	34	P	Infiltrating ductal carcinoma
	F	77	9	P	Infiltrating ductal carcinoma

Present case	F	49	0	C	Recurrent metastatic breast cancer

F: female, patient age: age at concurrent disease diagnosis, interval b/t diseases: interval between both diseases (sarcoidosis and breast cancer).

*∗*P: **preceded** breast cancer diagnosis; C: occurred **concomitantly** with breast cancer diagnosis; A: occurred **after** breast cancer diagnosis.
